# A VISION Substudy of Reader Agreement on ^68^Ga-PSMA-11 PET/CT Scan Interpretation to Determine Patient Eligibility for ^177^Lu-PSMA-617 Radioligand Therapy

**DOI:** 10.2967/jnumed.122.265077

**Published:** 2023-08

**Authors:** Phillip H. Kuo, Don C. Yoo, Ryan Avery, Marc Seltzer, Jeremie Calais, James Nagarajah, Wolfgang A. Weber, Wolfgang P. Fendler, Michael S. Hofman, Bernd J. Krause, Marcia Brackman, Euloge Kpamegan, Samson Ghebremariam, Taylor Benson, Ana M. Catafau, Ayse T. Kendi

**Affiliations:** 1 University of Arizona, Tucson, Arizona;; 2 Warren Alpert Medical School of Brown University, Providence, Rhode Island;; 3 Northwestern University, Evanston, Illinois;; 4 Geisel School of Medicine at Dartmouth, Hanover, New Hampshire;; 5 Ahmanson Translational Theranostics Division, Department of Molecular and Medical Pharmacology, UCLA, Los Angeles, California;; 6 Department of Medical Imaging, Radboud University Medical Center, Nijmegen, The Netherlands;; 7 TUM School of Medicine, Technical University of Munich, Munich, Germany;; 8 Department of Nuclear Medicine, University of Duisburg–Essen and German Cancer Consortium–University Hospital Essen, Essen, Germany;; 9 Cancer Imaging, Prostate Theranostics and Imaging Centre of Excellence, Peter MacCallum Cancer Centre, Melbourne, Victoria, Australia;; 10 Sir Peter MacCallum Department of Oncology, University of Melbourne, Melbourne, Victoria, Australia;; 11 Rostock University Medical Center, Rostock, Germany;; 12 Novartis Pharmaceuticals Corporation, Indianapolis, Indiana;; 13 Novartis Pharmaceuticals Corporation, East Hanover, New Jersey;; 14 Novartis Pharmaceuticals Corporation, St. George, Utah;; 15 Advanced Accelerator Applications, Geneva, Switzerland; and; 16 Mayo Clinic, Rochester, Minnesota

**Keywords:** PSMA, prostate cancer, PET/CT

## Abstract

[ ^68^Ga]Ga-PSMA-11 ( ^68^Ga-PSMA-11) is used to identify prostate-specific membrane antigen (PSMA)–positive tumors on PET scans. In the VISION study, ^68^Ga-PSMA-11 was used to determine the eligibility of patients with metastatic castration-resistant prostate cancer for treatment with [^177^Lu]Lu-PSMA-617 (^177^Lu-PSMA-617), based on predefined read criteria. This substudy aimed to investigate the interreader variability and intrareader reproducibility of visual assessments of ^68^Ga-PSMA-11 PET/CT scans using the VISION read criteria and evaluate the agreement between read results for this and the VISION study. **Methods:** In VISION, ^68^Ga-PSMA-11 PET/CT scans were centrally read as inclusion cases if they had at least 1 PSMA-positive lesion and no PSMA-negative lesions that fulfilled the exclusion criteria. In this substudy, 125 PET/CT scans (75 inclusion and 50 exclusion cases) were randomly selected from VISION and retrospectively assessed by 3 independent central readers. A random subset of 20 cases (12 inclusion and 8 exclusion cases) was recoded for assessment of intrareader reproducibility. Classification of cases as inclusion or exclusion cases was based on the VISION read criteria. Overall interreader variability was assessed by Fleiss κ-statistics, and pairwise variability and intrareader reproducibility were assessed by Cohen κ-statistics. **Results:** For interreader variability, the readers agreed on 77% of cases (overall average agreement rate, 0.85; Fleiss κ, 0.60 [95% CI, 0.50–0.70]). The pairwise agreement rate was 0.82, 0.88, and 0.84, and the corresponding Cohen κ was 0.54 (95% CI, 0.38–0.71), 0.67 (95% CI, 0.52–0.83), and 0.59 (95% CI, 0.43–0.75), respectively. For intrareader reproducibility, the agreement rate was 0.90, 0.90, and 0.95, and the corresponding Cohen κ was 0.78 (95% CI, 0.49–0.99), 0.76 (95% CI, 0.46–0.99), and 0.89 (95% CI, 0.67–0.99), respectively. The number of actual VISION inclusion cases out of the total number of cases scored as inclusion in this substudy was 71 of 93 (agreement rate, 0.76; 95% CI, 0.66–0.85) for reader 1, 70 of 88 (0.80; 0.70–0.87) for reader 2, and 73 of 96 (0.76; 0.66–0.84) for reader 3. All readers agreed on 66 of 75 VISION inclusion cases. **Conclusion:** Moderate-to-substantial interreader agreement and substantial-to-almost perfect intrareader reproducibility for ^68^Ga-PSMA-11 PET/CT scan assessment using the VISION read criteria were observed. The read rules applied in VISION can be readily learned and demonstrate good reproducibility.

## Visual Abstract

**Figure:**
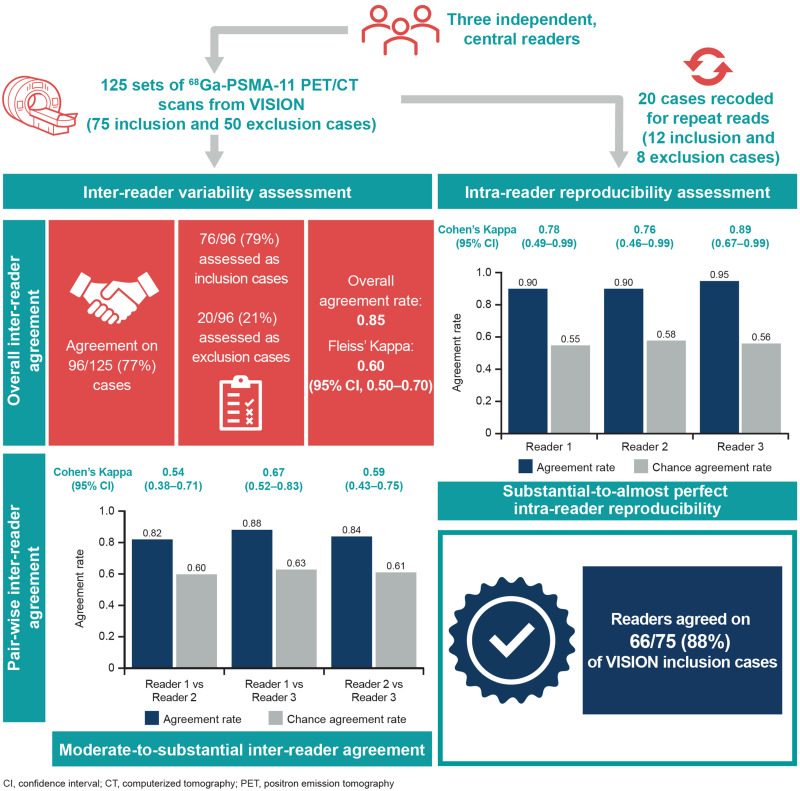

Prostate-specific membrane antigen (PSMA) is a transmembrane glutamate carboxypeptidase that is highly expressed in prostate cancer cells, with limited expression in non-prostate-cancer cells (1–3). This makes PSMA an important actionable theranostic target for patients with prostate cancer.

[^68^Ga]Ga-PSMA-11 (also known as ^68^Ga-PSMA-11) is an approved radioligand imaging agent used to identify PSMA-positive tumors on PET scans. In the pivotal phase 3 VISION study, ^68^Ga-PSMA-11 imaging was used to determine the eligibility of patients with metastatic castration-resistant prostate cancer (mCRPC) for radioligand therapy with [^177^Lu]Lu-PSMA-617 (also known as ^177^Lu-PSMA-617), based on predefined read criteria (4). These ^68^Ga-PSMA-11 PET/CT read rules were intended to select patients who were most likely to benefit from ^177^Lu-PSMA-617 in the VISION trial, following a population enrichment approach (5). VISION read rules were also designed to reduce future issues with reimbursement in using both ^18^F-FDG and PSMA PET scans (5).

There is reported evidence on the reliability of ^68^Ga-PSMA-11 PET scan reads in identifying PSMA-positive lesions across a range of diagnostic evaluation criteria and prostate cancer populations (6–11). Of note, the ProPSMA phase 3 study in the setting of staging demonstrated high reporter agreement between local and central review for ^68^Ga-PSMA-11 PET/CT, with κ-values of 0.87 for nodal and 0.88 for distant metastases (12). However, the reliability of read rules to establish the eligibility of patients with mCRPC for treatment with ^177^Lu-PSMA-617 in the VISION trial is yet to be determined.

In this independent VISION substudy, we aimed to assess the robustness of read rules used for scan interpretation in the VISION study. Specifically, we investigated the interreader variability and intrareader reproducibility of visual assessments of ^68^Ga-PSMA-11 PET/CT scans using the VISION read criteria for ^177^Lu-PSMA-617 therapy eligibility.

## MATERIALS AND METHODS

### Overview and Objectives

VISION was an open-label, international, randomized, phase 3 trial investigating the efficacy and safety of ^177^Lu-PSMA-617 in patients with progressive PSMA-positive mCRPC, previously treated with at least 1 androgen receptor pathway inhibitor and 1–2 taxane regimens. Details of the study design have been published elsewhere (4). This retrospective, independent, masked VISION substudy aimed to assess the variability across different readers (interreader variability) and the variability between different reads performed by the same reader (intrareader reproducibility) of ^68^Ga-PSMA-11 PET/CT scans, based on the VISION read rules used to determine patient eligibility for ^177^Lu-PSMA-617 therapy in the VISION study. The results from the eligibility determination in this reader agreement VISION substudy were also compared with the original eligibility results from the VISION study. Reader training, proficiency testing, and independent masked reads were conducted virtually on May 9–11, 2020.

### VISION ^68^Ga-PSMA-11 PET/CT Read Rules

In VISION, ^68^Ga-PSMA-11 scans were centrally read by 1 reader from a pool of 3 board-certified nuclear medicine physicians/radiologists. Readers were trained in person on the VISION read rules. VISION ^68^Ga-PSMA-11 PET/CT read rules have been reported and discussed in detail elsewhere (4*,*^5^). Briefly, patients with mCRPC with at least 1 PSMA-positive lesion identified by ^68^Ga-PSMA-11 PET/CT and no PSMA-negative lesion fulfilling the exclusion criteria were enrolled in the study, provided all other inclusion criteria were met (4). PSMA-positive lesions, of any size and present in any organ system, were identified first. These lesions were defined as those that had uptake greater than observed in the liver by visual assessment. PSMA-negative lesions were defined as those that had activity equal to or less than observed in the liver by visual assessment. Patients were excluded if one or more PSMA-negative lesions fulfilled the following size criteria measured on diagnostic imaging: lymph node at least 2.5 cm in short-axis diameter anywhere in the body, bone metastasis with soft-tissue component at least 1 cm in short-axis diameter, or solid-organ metastasis at least 1 cm in short-axis diameter (Supplemental Fig. 1; supplemental materials are available at http://jnm.snmjournals.org).

### Readers and Reader Training

Three independent central readers, each from a different institution, who were not previously involved in VISION ^68^Ga-PSMA-11 PET scan reads were asked to participate in this substudy. Readers were U.S. board-certified nuclear medicine physicians from different institutions; 2 readers were dual board-certified in radiology. Readers were experienced in reading PET/CT scans but not in reading ^68^Ga-PSMA-11 PET/CT scans or with the VISION read rules. A nuclear medicine radiologist involved in the development of the VISION read criteria and training of the central readers for the VISION study was assigned as the trainer. Because of the coronavirus disease 2019 pandemic, the readers were trained virtually, via the Zoom teleconference platform. The readers were guided through an approved independent-review training manual (developed by Invicro and Advanced Accelerator Applications), image software, the basics of PSMA PET/CT interpretation, and the VISION PET/CT scan read criteria. After completion of the training session, the readers were required to correctly assess at least 80% of 10 allocated training cases. The training cases were scored in a similar manner to the actual masked read to allow readers to familiarize themselves with the software and imaging evaluation. A reader with a score of below 80% would be provided with additional training and be reassessed for proficiency.

### Scan Selection and Coding

A random generator was used to select a total of 125 ^68^Ga-PSMA-11 PET scans and corresponding diagnostic CT/MRI scans from VISION to obtain a predetermined number of 75 inclusion cases (60%; patient enrolled) and 50 exclusion cases (40%; screen failure). These percentages intentionally deviated from the approximately 85% of inclusion cases in VISION to allow for a more robust exclusion case sample size for the evaluation of interreader variability. A randomly selected subset of 20 cases (12 inclusion cases and 8 exclusion cases) was also recoded for the evaluation of intrareader reproducibility. Scans for the 125 cases, plus 20 recoded repeats, and an additional 29 reader training cases were uploaded by the Invicro Image Management team to the Imaging Picture Archiving and Communication System, version 2.03. Digital Imaging and Communications in Medicine tags were modified with new patient identification randomization codes, and scans were uploaded to the mint Lesion software application (Mint Medical Inc.). All codes and files were reviewed and verified by Invicro, and scans were evaluated for anatomic coverage and quality.

### Independent Masked Read

#### Conduct

The readers could seek assistance on case loading and assessment recording from a proctor via Zoom; proctors were not able to comment on the assessments. The readers were provided with a list of PET/CT scans in a predefined read order that was unique to each reader. All cases, including the 20 recoded repeat cases, were read independently for 3 consecutive days, for approximately 8 h per day. The readers were allowed to take breaks whenever they wanted and were unaware of the patient data and each other’s results.

#### Assessment

The readers recorded their visual assessment of each scan on an electronic case report form and assessed whether a case was considered an inclusion or exclusion case for VISION enrollment (Supplemental Fig. 2).

### Statistical Analyses

Interreader variability was assessed by Fleiss κ-statistics, and interpretation was based on the Landis and Koch scale, whereby values of less than 0.00 were defined as poor disagreement, 0.00–0.20 as slight agreement, 0.21–0.40 as fair agreement, 0.41–0.60 as moderate agreement, 0.61–0.80 as substantial agreement, and 0.81–1.00 as almost perfect agreement (13*,*^14^). An overall average agreement rate (Pbar) for the Fleiss κ-analysis was calculated as the average agreement rate across the 3 readers for each of the 125 cases. Pairwise variability and intrareader reproducibility were assessed by Cohen κ-statistics. The agreement rate between independent reads in the substudy and VISION eligibility reads was calculated as the percentage of inclusion cases in VISION compared with cases assessed as inclusion by each independent reader. Statistical analyses were performed by an external consultant designated by Advanced Accelerator Applications.

### Study Oversight

VISION was registered on ClinicalTrials.gov (NCT03511664) and was conducted in accordance with the principles of the Declaration of Helsinki, the International Conference on Harmonization Good Clinical Practice, and any applicable local regulations. All patients in the pivotal study provided written informed consent before enrollment, and independent ethical review boards approved the VISION study protocol. This substudy was conducted by Invicro and funded by Advanced Accelerator Applications, a Novartis Company.

## RESULTS

### Conduct

Day 1 consisted of 4 h of reader training, followed by 2 h for the assessment of the 10 allocated training cases and 1.5 h for actual read time. Days 2 and 3 consisted of 7.5 h each for actual read time. The average number of cases read per hour was 8.8.

### Reader Proficiency Testing

After completion of the training session, all 3 readers scored 80% or higher in the correct assessment of the 10 training cases and required no further training.

### Interreader Variability

The readers agreed on the assessment of 96 of 125 cases (77%), of which 76 (79%) were scored as inclusion cases and 20 (21%) were scored as exclusion cases (Table 1). The agreement rates for inclusion and exclusion cases were 88% and 60%, respectively. The Pbar between readers was 0.85; the Fleiss κ was 0.60 (95% CI, 0.50, 0.70), representing moderate-to-substantial interreader agreement.

**TABLE 1.** Concordance Combinations Among 3 Readers (125 Cases)

Reader outcomes			Results	
Reader 1	Reader 2	Reader 3	Frequency (*n*)	Proportion (%)
Inclusion	Inclusion	Inclusion	76	61
Inclusion	Inclusion	Exclusion	3	2
Inclusion	Exclusion	Inclusion	11	9
Inclusion	Exclusion	Exclusion	3	2
Exclusion	Inclusion	Inclusion	6	5
Exclusion	Inclusion	Exclusion	3	2
Exclusion	Exclusion	Inclusion	3	2
Exclusion	Exclusion	Exclusion	20	16

**FIGURE 1.**
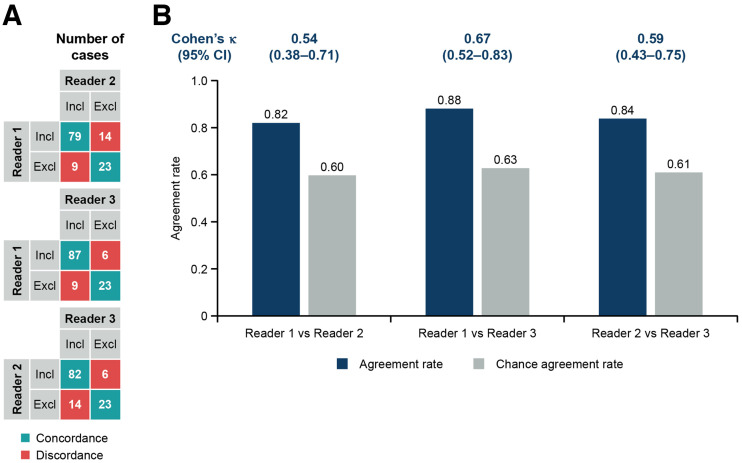
Pairwise interreader agreement (125 cases). (A) Pairwise concordance combinations. (B) Cohen κ-analysis. κ-statistic is calculated as (observed agreement – chance agreement)/(1 – chance agreement). Chance agreement is probability that readers randomly agree. Excl = cases assessed as exclusion; Incl = cases assessed as inclusion.

The pairwise agreement rate between readers 1 and 2, readers 1 and 3, and readers 2 and 3, was 0.82, 0.88, and 0.84, respectively; the corresponding Cohen κ was 0.54 (95% CI, 0.38, 0.71), 0.67 (95% CI, 0.52, 0.83), and 0.59 (95% CI, 0.43, 0.75), respectively, representing moderate-to-substantial agreement between all 3 pairs of readers (Fig. 1).

**FIGURE 2.**
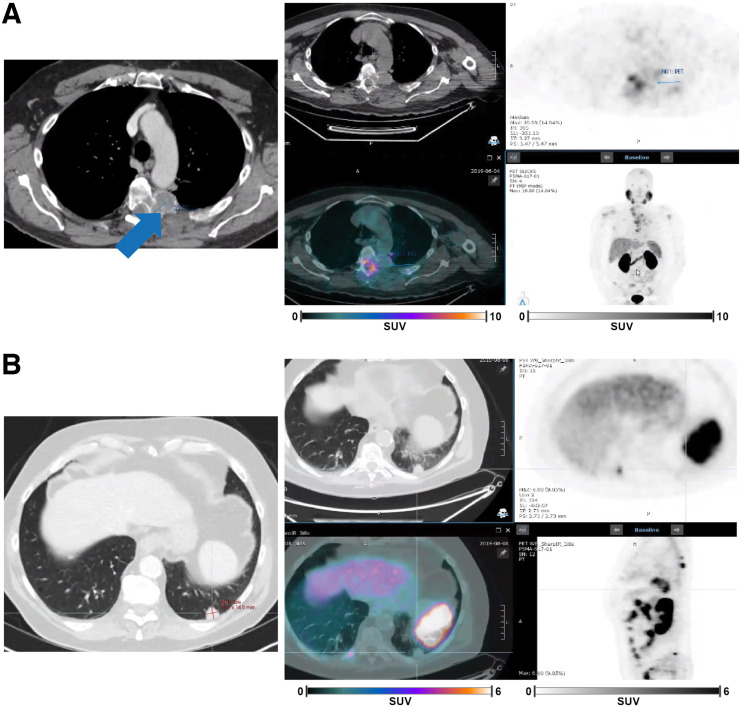
Illustrative PSMA PET/CT and diagnostic CT scans for discordant cases. (A) In the left panel, transaxial slice from diagnostic CT with intravenous contrast at level of aortic arch shows left thoracic vertebral metastasis (arrow) with soft-tissue component greater than 1 cm in short axis. In the right panel, PSMA PET/CT transaxial and maximum-intensity-projection images from mint Lesion were captured from annotations by 2 of 3 readers who correctly identified PSMA-negative vertebral lesion. (B) In the left panel, transaxial slice from diagnostic CT with intravenous contrast medium at level of lung bases demonstrates left lung nodule measuring 1.5 cm in short axis. In the right panel, PSMA PET/CT transaxial and sagittal PET images from mint Lesion were captured from annotations by 2 of 3 readers who classified lung nodule as PSMA-negative. One reader may not have identified this lesion as PSMA-negative since other nodules (not shown) were PSMA-positive.

Of the 29 of 125 (23%) discordant cases, some of which belonged to multiple regions, the readers differed in their assessment of lymph node (6 cases) and of bone metastasis, liver, or cases with no positive lesion (5 each). There were 9 discordant cases that included lesions in the prostate/urinary bladder (*n* = 4), lung (*n* = 2), adrenal gland (*n* = 2), and kidney (*n* = 1). Illustrative examples of PET/CT scans for discordant exclusion cases are shown in Figure 2, whereby one reader did not see a bone lesion with a PET-negative soft-tissue component in a patient with multiple positive PET-positive lesions, and another did not see a PET-negative lung lesion among multiple PET-positive lesions.

### Intrareader Reproducibility

**FIGURE 3.**
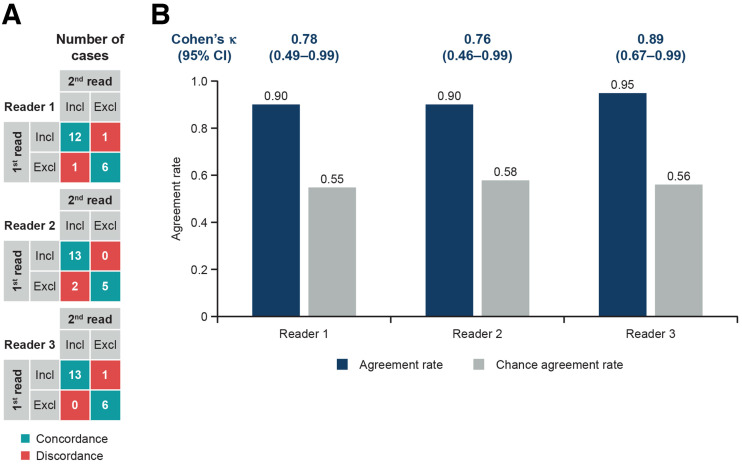
Intrareader agreement (20 cases). (A) Concordance combinations. (B) Cohen κ-analysis. κ-statistic is calculated as (observed agreement – chance agreement)/(1 – chance agreement). Chance agreement is probability that readers randomly agree. Excl = cases assessed as exclusion; Incl = cases assessed as inclusion.

For the 20 recoded cases that were read twice by each reader, the agreement rate was 0.90, 0.90, and 0.95 for readers 1, 2, and 3, respectively. The corresponding Cohen κ was 0.78 (95% CI, 0.49, 0.99), 0.76 (95% CI, 0.46, 0.99), and 0.89 (95% CI, 0.67, 0.99), respectively, representing substantial -to-almost perfect agreement for all 3 readers (Fig. 3).

### Agreement Rates with VISION Eligibility Read Results

**FIGURE 4.**
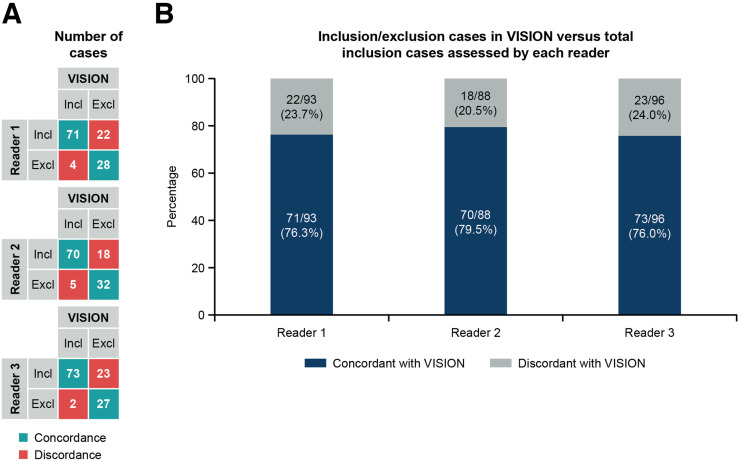
Agreement between substudy read results and VISION eligibility read results (125 cases). (A) Concordance combinations between individual readers in substudy and eligibility assessments in VISION. (B) Proportion of actual inclusion and exclusion cases in VISION among total number of inclusion cases assessed by reader in this substudy. Excl = cases assessed as exclusion; Incl = cases assessed as inclusion.

Read results from this study were compared with the VISION eligibility read results used to determine patient enrollment. The agreement rate, defined as the proportion of actual inclusion cases in VISION, out of the number of cases scored as inclusion by each reader in the reader agreement study, was 0.76 (95% CI, 0.66, 0.85), 0.80 (95% CI, 0.70, 0.87), and 0.76 (95% CI, 0.66, 0.84) (Fig. 4). Of the 75 inclusion cases from VISION, there was complete agreement among the 3 readers on the assessment of 66 (88%) cases as inclusion cases. The remaining 9 (12%) cases that the readers disagreed on were unique to each reader. Better concordance was observed for inclusion cases than for exclusion cases for all 3 readers.

## DISCUSSION

To date, the reported reader agreement on ^68^Ga-PSMA-11 PET scans has been based on diagnostic criteria (6–11*,*^15^). In this independent substudy of the phase 3 VISION study, we assessed the robustness of the VISION read criteria for enrollment of patients with mCRPC, previously treated with at least 1 androgen receptor pathway inhibitor and 1–2 taxane regimens, for treatment with ^177^Lu-PSMA-617 in VISION. According to the Landis and Koch scale, interpretation of ^68^Ga-PSMA-11 PET/CT scans using the VISION read criteria showed moderate-to-substantial interreader agreement and substantial-to-almost perfect intrareader reproducibility. Substantial agreement between read results in this substudy and the VISION study was also observed. Overall, agreement rates were consistently higher for inclusion cases than for exclusion cases.

In VISION, the CT scan was used to identify more aggressive lesions (i.e., anatomically measurable lesions according to the read rules for exclusion) and visual assessments were used instead of quantitative assessments for PSMA positivity criteria, using liver uptake as a reference organ (4). Using this approach, the need for an additional ^18^F-FDG PET/CT scan or PET quantification was avoided. Overall, the moderate-to-substantial level of interreader agreement (Pbar, 0.85; Fleiss κ, 0.60 [95% CI, 0.50, 0.70]) in ^68^Ga-PSMA-11 PET/CT scan interpretation in this VISION substudy was similar to what has been previously reported, although it should be noted that these studies use different study criteria and are in different disease settings (Supplemental Table 1). Intrareader reproducibility for repeated reads by the same reader was 90%–95%, with a corresponding Cohen κ of 0.76–0.89, showing excellent agreement for all 3 readers. These results indicate that the reproducibility of read rules for patient selection is high. To our knowledge, this substudy is the first to determine reader agreement in the visual interpretation of ^68^Ga-PSMA-11 PET/CT scans in patients with mCRPC for eligibility assessment of ^177^Lu-PSMA-617, in the context of applying a population enrichment approach within a clinical trial. In clinical practice, selection of patients for treatment with ^177^Lu-PSMA-617 may require multidisciplinary consultation for borderline or difficult-to-interpret scans by a single reader (16).

Comparison of read results in this study and VISION eligibility read results demonstrated an agreement rate of between 76% and 80%, with better concordance among inclusion cases. All readers agreed on the assessment of 66 of 75 VISION inclusion cases. The remaining cases, which the readers did not agree on, were unique, and although these were assessed as inclusion cases in VISION, there is a possibility that the central reader was incorrect in their assessment.

There were several study limitations that could have resulted in variability between readers and between VISION read results. First, in the VISION study, the readers were trained in person. The same was initially planned for this substudy; however, because of the coronavirus disease 2019 pandemic, training, proficiency testing, and the retrospective reading of PET/CT scans were conducted virtually. Mitigation strategies were implemented to reduce potential study variability, including the provision of identical multimonitor workstations and pretesting of the transfer of data-heavy PET/CT images using residential Internet service. However, caveats to the virtual approach included potentially less comprehensive training and technical issues such as delays in high-resolution image display and scrolling, which may have led to reader fatigue. Second, and leading on from this, reads in VISION were performed for just a few cases per session during VISION enrollment, but in this study, the readers assessed an average of 8.8 cases per hour. Therefore, another cause of reader fatigue in this study may have resulted from the 8-h sessions over 3 consecutive days, which may have also affected visual search patterns (17). This aspect could particularly affect the search for CT-measurable but PSMA-negative lesions, which may account for the higher variability found in exclusion cases. Third, the readers had little or no experience with PSMA PET/CT scans, which is different from the current standard of care now that PSMA PET agents are U.S. Food and Drug Administration-approved and in wider use. Fourth, unlike what we expect for implementation in standard practice, no prior imaging or reports were available, which may have complicated the interpretation of the true metastatic nature of lesions and also identification of all relevant lesions on CT.

A higher agreement rate was observed between inclusion cases than between exclusion cases. Overall, 40% of cases were exclusion cases, compared with the 12.6% in VISION that did not meet the imaging criteria. A higher proportion of exclusion cases was included in this study to support statistical analyses; however, challenges associated with the interpretation of exclusion cases may have also contributed to increased interreader variability. For example, the identification of negative lesions requires readers to search for anatomic lesions on CT/MRI scans with no or low corresponding image tracer uptake on PET. It is generally easier to identify hot lesions than cold ones. Therefore, oversight of an anatomic lesion on CT/MRI may lead to misclassification of an exclusion case as an inclusion case. In addition, lack of access to prior diagnostic imaging or reports may have been a limiting factor in identifying lesions and characterizing whether lung and adrenal nodules, for example, were truly metastatic. Finally, reader variability associated with the identification of different lesion types such as PSMA-negative lymph nodes and bone metastases with soft-tissue components—because of more challenging tumors with necrotic components, for example—could have been another limiting factor. Indeed, 11 of 29 discordant cases in this substudy were attributed to the assessment of lymph node and bone metastases.

To minimize discordance in case assessment in clinical practice, careful reading of the diagnostic CT scan using region-anatomic and organ-specific windows is recommended. The often very high uptake of metastatic lesions in mCRPC can tempt readers to view the PET imaging at too wide a window. Since the threshold for PSMA positivity or negativity is the liver, active windowing of the PET imaging, with the liver initially placed in the middle of the window, is recommended. Specifically for extensive PSMA-positive adenopathy, focal areas of decreased uptake should be carefully assessed for negative nodes. For prostate bed or urinary bladder assessments, viewing in coronal and sagittal planes and multiple window intensities is recommended. For aggressive disease invading the urinary bladder and surrounding structures, the CT scan is critical since uptake by the disease may be similar to the urinary activity. For bone metastases, CT scans should be read on both bone and soft-tissue windows, and PET and PET/CT images should be assessed for mild activity outside the margins of the cortical bone. For liver metastases, careful reading of the CT scan is essential to identify lesions since PSMA-negative metastases will often be invisible against the background of normal liver uptake. Viewing the region of the metastasis in multiple axes on the PET scan is suggested given the frequency of motion artifacts. Finally, one should recognize the critical difference between this read paradigm and the typical use of PSMA PET for staging or recurrence. For approximately 95% of cases, the goal is not to identify all sites of PSMA-positive disease but rather to ensure that any PSMA-negative lesion meeting the size criteria is identified. It is inherently easier to see PSMA-positive than PSMA-negative disease and thus fall into the trap of “satisfaction of search.” Therefore, the reader needs to tune out the often numerous PSMA-positive lesions and tune in to finding lesions at or below the level of liver uptake—a task that may be feasible only by first identifying the metastasis on CT.

## CONCLUSION

This VISION substudy demonstrated moderate-to-substantial interreader agreement and substantial-to-almost perfect intrareader agreement on visual assessment of ^68^Ga-PSMA-11 PET/CT scans, according to predefined VISION rules. The read rules used in VISION to determine patient eligibility for treatment with ^177^Lu-PSMA-617 were readily learned and demonstrated good reproducibility among independent reviewers, despite the limitations of this substudy.

## DISCLOSURE

This work was funded by Advanced Accelerator Applications, a Novartis Company. Phillip H. Kuo was an employee of Invicro LLC and reports consulting or speaker fees from Amgen, Bayer AG, Chimerix, Eisai, Fusion Pharma, GE Healthcare, Invicro, Novartis, and UroToday and research funding from Blue Earth Diagnostics and GE Healthcare. Don C. Yoo and Ryan Avery report consulting fees for Invicro LLC. Jeremie Calais reports prior consulting activities for Advanced Accelerator Applications, Astellas, Blue Earth Diagnostics, Curium Pharma, DS Pharma, EXINI, GE Healthcare, IBA RadioPharma, Isoray, Janssen Pharmaceuticals, Lightpoint Medical, Lantheus, Monrol, Novartis, POINT Biopharma, Progenics, Radiomedix, Sanofi, and Telix Pharmaceuticals. James Nagarajah reports consulting fees from Curium Pharma and POINT Biopharma, speaker fees from Bayer AG, and research funding from Advanced Accelerator Applications. Wolfgang A. Weber reports consulting or speaker/advisory fees from Advanced Accelerator Applications, Bayer AG, Blue Earth Diagnostics, GSK, ITM, Pentixapharm, Rayzebio, Reflexion, and Vida Ventures and research funding from BMS, Imaginab, Ipsen, Nuclidium, Piramal, and TRIMT. Wolfgang P. Fendler reports consulting fees or speaker fees from Advanced Accelerator Applications, Bayer AG, Calyx, Janssen Pharmaceuticals, and Parexel and research funding from Bayer AG and Sofie Biosciences. Michael S. Hofman reports consulting fees from Astellas, AstraZeneca, Janssen Pharmaceuticals, Merck/MSD, Mundipharma, and POINT Biopharma and research funding from Advanced Accelerator Applications. Bernd J. Krause reports consulting fees from Advanced Accelerator Applications, Bayer AG, and ITM and research funding from Advanced Accelerator Applications. Marcia Brackman, Euloge Kpamegan, Samson Ghebremariam, and Taylor Benson are employees and stockholders of Novartis, and Ana M. Catafau is an employee and stockholder of Advanced Accelerator Applications. Ayse T. Kendi reports consulting fees and research funding from Novartis. Under the direction of the authors, Sarah Sabir from Oxford PharmaGenesis, Oxford, U.K., provided medical writing support, which was funded by Novartis, in accordance with Good Publication Practice 3 guidelines (https://www.ismpp.org/gpp3). No other potential conflict of interest relevant to this article was reported.

## Notes

KEY POINTS **QUESTION:** This study investigated the interreader variability and intrareader reproducibility of visual assessment of ^68^Ga-PSMA-11 PET/CT scans using the VISION read criteria and evaluated agreement between read results for this and the VISION study. **PERTINENT FINDINGS:** This was a retrospective, masked VISION substudy in which 3 independent readers assessed 125 PET/CT scans from VISION, according to the VISION read criteria. The 3 readers agreed on the assessment of 77% of cases, showing moderate-to-substantial interreader agreement (Fleiss κ, 0.60 [95% CI, 0.50, 0.70]). The intrareader reproducibility of ^68^Ga-PSMA-11 PET/CT scan assessment was substantial to almost perfect (Cohen κ, 0.78 [95% CI, 0.49, 0.99], 0.76 [95% CI, 0.46, 0.99], and 0.89 [95% CI, 0.67, 0.99]). Comparison of the reader results in this study and the VISION eligibility read results demonstrated an agreement rate of between 76% and 80%. **IMPLICATIONS FOR PATIENT CARE:** Visual assessment of ^68^Ga-PSMA-11 PET/CT scans using the VISION read criteria to enrich the patient population in VISION can be readily learned and demonstrates good reproducibility. In clinical practice, variations of this approach for patient selection may apply.
